# Association between fatty acids and the risk of impaired glucose tolerance and type 2 diabetes mellitus in American adults: NHANES 2005−2016

**DOI:** 10.1038/s41387-023-00236-4

**Published:** 2023-05-01

**Authors:** Xiaoqiong Zhu, Liu Chen, Jiansheng Lin, Mingqin Ba, Junqiu Liao, Ping Zhang, Cunxi Zhao

**Affiliations:** 1 Department of Nutrition, School of Public Health, Anhui Medical University, Anhui Hefei, 230032 PR China; 2 Jieyang Center for Disease Control and Prevention, Guangdong, Jieyang 522000 PR China

**Keywords:** Risk factors, Epidemiology

## Abstract

**Background:**

Fatty acids (FAs) play a major role in regulating insulin sensitivity. However, owing to dietary quantitative tools, it has been challenging to study the dietary FAs in previous studies. There is a lack of knowledge regarding the associations between dietary FAs and the risk of impaired glucose tolerance (IGT) and type 2 diabetes mellitus (T2DM).

**Methods:**

Dietary FAs, adjustment of variables including age, sex, race, educational level, poverty to income ratio, body mass index, smoking, hypertension, physical activity, and diabetes data were extracted from the National Health and Nutrition Examination Survey 2005–2016. A multivariate logistic regression model was used to determine the associations between FA intake and the risk of IGT and T2DM.

**Results:**

This serial cross-sectional study included 9082 samples. After adjusting all the variables, a negative correlation was observed between total saturated FA and the risk of IGT (OR = 0.991, 95% (CI): 0.985–0.998, *P* = 0.024). Total FA at quintile 4 was negatively correlated with T2DM (OR = 0.714, 95% CI: 0.532–0.959, *P* = 0.025) compared with quintile 1. Factor analysis identified four factors of which F4 was negatively associated with the risk of T2DM (OR = 0.824, 95% CI: 0.715–0.949, *P* = 0.029). Based on this factor, we identified an unsaturated FA signature (*n* = 4 FAs, including octadecenoic acid (18:1), octadecadienoic acid (18:2), octadecatrienoic acid (18:3), and eicosenoic acid (20:1)).

**Conclusions:**

Several unsaturated FAs with high proportions in natural oils may reduce the risk of T2DM.

## Introduction

It is essential to control type 2 diabetes mellitus (T2DM), a serious public health threat that has doubled over the past few decades and is projected to rise by more than 100 million cases in the coming decade [1]. T2DM development is related to genetics, lifestyle, and dietary patterns [2]. Dietary fat, an integral part of the daily diet, has been implicated in T2DM [3]. However, whether a high-fat diet increases the risk of impaired glucose tolerance (IGT) and T2DM and specific types of fatty acids (FAs), which are risk factors or protective factors for T2DM remains controversial.

A recent study has revealed that a high-fat diet can augment intestinal fructose metabolism and its derived glycerol, causing chronic damage to islet cells [4]. The function of FAs cannot be generalized. Saturated FAs (SFAs) are usually considered to be pro-inflammatory molecules; in addition to exhibiting lipid-lowering effect, omega-3 FAs (such as eicosatetraenoic acid and docosahexaenoic acid) are also recognized as anti-inflammatory molecules to reduce the inflammatory process [5]. Ultimately, different FAs play distinct roles in the development of inflammation-mediated insulin resistance. Long-chain SFA intake, particularly palmitic acid, can trigger insulin resistance in pancreatic β-cells by activating c-Jun N-terminal kinase expression [6]. However, long-chain unsaturated FAs promote the secretion of glucagon-like peptide-1 by activating the expression of the G-protein-coupled receptor, thus, increasing circulating insulin [7].

Although these studies indicate that FAs play a key role in the occurrence and development of diabetes, owing to dietary quantitative tools, it has been difficult to study the association between dietary FA subtypes and the risk of IGT and T2DM in previous studies. Free FAs in the blood do not provide a reasonable guide to dietary recommendations for diabetes. Based on the National Health and Nutrition Examination Survey (NHANES) database, we assessed the association between dietary FAs and the risk of IGT and T2DM. We focused not only on the correlation between dietary FAs and the risk of IGT and T2DM but also on whether these effects are dose-dependent. We can better understand the relationship between FAs and T2DM and lay the groundwork for further prospective studies and biological mechanisms by narrowing the type and dose range of FAs.

## Materials and methods

### Study population

The NHANES, an underway cross-sectional research program, authorized by the National Center for Health Statistics Research Ethics Review Board, is representative samples of American populations. It uses a stratified multi-stage probability sample to select a nationally representative sample of civilians. We used all the available public domain data from six cycles of NHANES 2005−2016 for this research. Since every participant had a specific identification number, we combined the data. To remove the Neyman bias, our study excluded participants whose dietary structure changed under the guidance of doctors and nurses owing to diagnosed diabetes [8]. Participants were further excluded if their fasting plasma glucose and 2-hour oral glucose tolerance test results were incomplete or missing key covariates. In this study, there were ultimately 9,082 participants who made up the analytical sample. Previous studies have provided more comprehensive information on blood biochemical indicators and data analysis [9, 10].

### Definitions of T2DM and IGT

T2DM and IGT were diagnosed following the World Health Organization: a fasting plasma glucose level of ≥7.0 mmol/L (126 mg/dL) or a 2-hour oral glucose tolerance level of ≥11.1 mmol/L (200 mg/dL) was defined as T2DM. A fasting plasma glucose level of <7.0 mmol/L (126 mg/dL) or 2-h oral glucose tolerance level of ≥7.8 and<11.1 mmol/L (140 and 200 mg/dL, respectively) was defined as IGT [11]. In addition to these, they are all normal glucose tolerance (NGT).

### FA intake

Dietary FAs and subtype intakes were estimated by two 24-hour recall interviews, which were collected in person in the Mobile Examination Center and then collected by telephone 3–10 days later. The daily dietary FA and subtype intakes were calculated based on the U.S. Department of Agriculture’s Dietary Research Food and Nutrition Database for Dietary Studies [12]. To fully utilize dietary data, we used the average of the data when the two 24-hour recall data were complete, single data if there were missing data, and excluded the participants when both data were missing.

### Covariates

Covariates that may influence the association between FA intake and T2DM risk were derived from direct interviews and medical center examinations, which included age, sex (male and female), race/ethnicity (Mexican American, other Hispanic, non-Hispanic white, non-Hispanic Black, and other races), educational level (less than 9th grade, 9–11th grade, high school graduate, some college//AA graduate, college graduate), poverty to income ratio (PIR), body mass index (BMI), smoking (Yes/No), hypertension (Yes/No), and physical activity (Yes/No) for this study. The classifications of covariates are depicted in Table 1.

**Table 1:** Sociodemographic characteristics of study participants: NGT versus IGT and T2DM^a,b^.

Sociodemographic characteristics	NGT^a^ (*N* = 6892)	IGT^a^ (*N* = 1434)	T2DM^a^ (*N* = 765)	*P* value^b^
Age(years)	44.19 ± 15.68	54.80 ± 16.12	59.32 ± 14.85	**<0.001**
Sex				NS
Male	3494 (50.7%)	718 (50.1%)	420 (54.9%)	
Female	3398 (49.3%)	716 (49.9%)	345 (45.1%)	
BMI (kg/m^2^)	27.48 ± 5.85	29.83 ± 6.61	30.70 ± 6.30	**<0.001**
Race/Ethnicity				NS
Mexican American	951 (13.8%)	232 (16.2%)	123 (16.1%)	
Other Hispanic	622 (9.0%)	131 (9.1%)	72 (9.4%)	
Non-Hispanic White	3335 (48.4%)	730 (50.9%)	399 (52.2%)	
Non-Hispanic Black	1330 (19.3%)	241 (14.9%)	119 (15.6%)	
Other Race	654 (9.5%)	127 (8.9%)	52 (6.8%)	
Education				**<0.001**
Less than 9th grade	411 (6.0%)	184 (12.8%)	96 (12.5%)	
9–11th grade	822 (12.8%)	199 (13.9%)	118 (15.4%)	
High school graduate	1536 (22.3%)	351 (24.5%)	226 (29.5%)	
Some college/AA degree	2148 (31.2%)	389 (27.1%)	205 (26.8%)	
College graduate	1915 (27.8%)	311 (21.7%)	120 (15.7%)	
Family PIR (%)	3.14 ± 1.62	3.00 ± 1.62	2.85 ± 1.61	**0.001**
Smoking				**<0.001**
Yes	1870 (27.1%)	273 (19.0%)	166 (21.7%)	
No	5022 (72.9%)	1161 (81.0%)	599 (78.3%)	
Hypertension				**<0.001**
Yes	1802 (26.1%)	706 (49.2%)	418 (54.6%)	
No	5090 (73.9%)	728 (50.8%)	347 (45.4%)	
Exercise				**<0.001**
Yes	2263 (32.8%)	256 (17.9%)	109 (14.2%)	
No	4629 (67.2%)	1178 (82.1%)	656 (85.8%)	

*BMI* body mass index, *PIR* poverty to income ratio.

^a^Data are mean and standard or percentage.

^b^*P* values corrected by FDR.

### Statistical analysis

To explain nationally representative estimates, the analyses were sampling weighted in this study. Normal distributions were presented as means standard deviation, otherwise, described by median and interquartile range. Categorical variables were described by percentage, which adopted the separate analysis of variance or Chi-square tests to compare the discrepancy among the NGT, IGT, and T2DM groups. Dietary FA intakes were divided into quartiles (Q), and Q1 as the reference. To identify a set of uncorrelated factors, we performed factor analysis with varimax (orthogonal) rotation. By reducing the number of latent variables (or dimensions), factor analysis identified factors that explain how the FA are related to one another. Factor loading greater than 0.4 was significant. Multivariate logistic regression analyses were applied to explore the correlation between FA intake and the risk of IGT and T2DM. Model 1 was not adjusted; Model 2 was adjusted for age and sex; Model 3 was further adjusted for race, BMI, education, PIR, smoking, hypertension, and exercise. All statistical analyses were conducted using R software version 4.0.2. A two-sided *P* < 0.05 was used to identify statistically significant results. Multiple comparisons used the Benjamini−Hochberg approach to control the false positive range.

## Results

Table 1 presented the demographic characteristics among NGT, IGT, and T2DM groups. A total of 9082 participants, 6892 participants with NGT, 765 participants with T2DM, and 1434 participants with IGT, were finally included in this study. Participants in T2DM and IGT groups tended to be older with larger BMI than those in the NGT group (*P* < 0.05). IGT and T2DM individuals had lower education, less smoking, and more hypertension and were less likely to exercise compared with NGT individuals (*P* < 0.05).

The average FA intakes and the difference between T2DM, IGT, and NGT groups are summarized in Table 2. The dietary intakes of total FA (TFA), total saturated FA (TSFA), monounsaturated FA (MUFA), and polyunsaturated FA (PUFA) were included in the study. Among them, the intakes of TFA, MUFA, and PUFA were statistically lower in IGT and T2DM groups than those in the NGT group (*P* < 0.05). The average intake of TSFA and SFA subtypes in the IGT group was significantly lower than that in T2DM and NGT groups, including butanoic acid (4:0), hexanoic acid (6:0), octanoic acid (8:0), decanoic acid (10:0), dodecanoic acid (12:0), tetradecanoic acid (14:0), hexadecanoic acid (16:0), and octadecanoic acid (18:0). Among MUFAs, hexadecenoic acid (16:1), octadecenoic acid (18:1), and eicosenoic acid (20:1) were statistically lower in T2DM and IGT groups than those in the NGT group. Among PUFAs, octadecadienoic acid (18:2) and octadecatrienoic acid (18:3) were statistically lower in T2DM and IGT groups than those in the NGT group. There was no statistical difference in the remaining FAs among the three groups.

**Table 2:** Different dietary FAs intakes between NGT, IGT and T2DM groups^a^.

FAs(g/d)	NGT (*n* = 6892)	IGT (*N* = 1434)	T2DM (*N* = 765)	*P*^a^
TFA	78.69 ± 38.79	72.31 ± 36.79	71.72 ± 38.62	**<0.001**
TSFA	25.78 ± 14.02	23.27 ± 13.15	23.68 ± 13.53	**<0.001**
4:0	0.53 ± 0.46	0.47 ± 0.43	0.50 ± 0.42	**<0.001**
6:0	0.31 ± 0.27	0.28 ± 0.26	0.29 ± 0.25	**<0.001**
8:0	0.26 ± 0.28	0.23 ± 0.23	0.24 ± 0.23	**<0.001**
10:0	0.47 ± 0.40	0.41 ± 0.37	0.43 ± 0.36	**<0.001**
12:0	0.80 ± 1.12	0.71 ± 1.07	0.72 ± 0.95	**0.014**
14:0	2.18 ± 1.62	1.90 ± 1.49	2.02 ± 1.48	**<0.001**
16:0	14.12 ± 7.47	12.8 ± 6.84	12.98 ± 7.12	**<0.001**
18:0	6.48 ± 3.62	5.87 ± 3.29	6.03 ± 3.38	**<0.001**
MUFA	28.34 ± 14.77	26.23 ± 13.91	25.92 ± 14.68	**<0.001**
16:1	1.17 ± 0.75	1.06 ± 0.68	1.10 ± 0.71	**<0.001**
18:1	26.56 ± 14.16	24.6 ± 13.08	24.4 ± 13.70	**<0.001**
20:1	0.29 ± 0.23	0.26 ± 0.23	0.25 ± 0.20	**<0.001**
22:1	0.04 ± 0.15	0.03 ± 0.13	0.04 ± 0.11	0.576
PUFA	17.73 ± 9.91	16.64 ± 9.51	15.94 ± 10.01	**<0.001**
n-6				
18:2	15.74 ± 8.99	14.73 ± 8.68	14.12 ± 8.94	**<0.001**
18:3	1.62 ± 1.06	1.52 ± 1.00	1.47 ± 0.99	**<0.001**
18:4	0.02 ± 0.05	0.02 ± 0.05	0.02 ± 0.05	0.083
20:4	0.15 ± 0.11	0.15 ± 0.12	0.14 ± 0.11	0.141
n-3				
20:5	0.04 ± 0.12	0.04 ± 0.13	0.04 ± 0.11	0.770
22:5	0.03 ± 0.04	0.02 ± 0.04	0.02 ± 0.05	0.132
22:6	0.08 ± 0.18	0.08 ± 0.20	0.08 ± 0.17	0.964

^a^*P* value corrected by FDR.

The correlation between FA intake and the risk of IGT and T2DM is indicated in Table 3. In model 1, a negative correlation was observed between dietary TFA and the risk of IGT (odds ratio [OR] = 0.996, 95% confidence interval [CI]: 0.994–0.999, *P* = 0.008). A negative correlation was also observed between TSFA and MUFA with the risk of IGT (OR = 0.989, 95% CI: 0.983–0.999, *P* < 0.001; OR = 0.992, 95% CI: 0.987–0.998, *P* = 0.013). Among SFAs, all SFAs were negatively correlated with IGT except for hexanoic acid (6:0) and dodecanoic acid (12:0). Among MUFAs, hexadecenoic acid (16:1) and octadecenoic acid (18:1) were negatively correlated with the risk of IGT (OR = 0.850, 95% CI: 0.762–0.948, *P* = 0.010; OR = 0.991, 95% CI: 0.985–0.997, *P* = 0.012). Among PUFA, only octadecatrienoic acid (18:3) was negatively correlated with the risk of IGT (OR = 0.903, 95% CI: 0.835–0.977, *P* = 0.021). FAs with statistical significance in model 1 were included in model 2 for analysis. In model 2 and model 3, TSFA, octanoic acid (8:0), decanoic acid (10:0), tetradecanoic acid (14:0), octadecanoic acid (18:0), and octadecatrienoic acid (18:3) were negatively associated with the risk of IGT. However, no significant association between FAs and T2DM was observed.

**Table 3:** Association between FAs and the risk of IGT and T2DM in NHANES 2013–2016^a,b^.

FAs(g/d)	IGT			T2DM		
	aOR^a^	95%CI	*P*^b^	aOR^a^	95%CI	*P*^b^
Model 1						
TFA	0.996	0.994–0.999	**0.008**	0.997	0.994–0.999	0.115
TSFA	0.989	0.983–0.995	**<0.001**	0.995	0.987–1.002	0.377
4:0	0.759	0.630–0.914	**0.010**	0.933	0.753–1.157	0.762
6:0	0.722	0.528–0.989	0.074	0.970	0.704–1.337	0.893
8:0	0.560	0.384–0.817	**0.010**	0.844	0.604–1.179	0.564
10:0	0.680	0.545–0.850	**0.005**	0.858	0.686–1.074	0.378
12:0	0.912	0.825–1.008	0.101	0.921	0.820–1.034	0.377
14:0	0.898	0.850–0.948	**<0.001**	0.971	0.911–1.033	0.575
16:0	0.979	0.968–0.990	**<0.001**	0.989	0.975–1.003	0.345
18:0	0.957	0.935–0.979	**<0.001**	0.985	0.957–1.013	0.539
MUFA	0.992	0.987–0.998	**0.013**	0.993	0.986–0.999	0.125
16:1	0.850	0.762–0.948	**0.010**	0.973	0.850–1.115	0.852
18:1	0.991	0.985–0.997	**0.012**	0.992	0.985–0.999	0.115
20:1	0.728	0.491–1.079	0.146	0.543	0.328–0.900	0.115
22:1	0.789	0.419–1.484	0.531	0.910	0.555–1.492	0.852
PUFA	0.992	0.984–1.001	0.101	0.984	0.972–0.995	0.069
n-6						
18:2	0.990	0.981–0.999	0.076	0.981	0.969–0.994	0.069
18:3	0.903	0.835–0.977	**0.021**	0.871	0.768–0.987	0.115
18:4	0.267	0.054–1.328	0.145	0.346	0.023–5.219	0.679
20:4	1.246	0.592–2.622	0.617	1.069	0.406–2.815	0.893
n-3						
20:5	0.855	0.401–1.825	0.717	1.163	0.475–2.851	0.852
22:5	0.212	0.011–4.032	0.366	0.731	0.018–29.015	0.893
22:6	0.994	0.632–1.562	0.979	1.156	0.648–2.061	0.843
Model 2						
TFA	0.998	0.995–1.000	0.081	0.998	0.996–1.001	0.664
TSFA	0.992	0.985–0.999	**0.047**	1.000	0.992–1.007	0.980
4:0	0.815	0.669–0.994	0.066	1.023	0.819–1.276	0.980
8:0	0.634	0.437–0.920	**0.047**	0.969	0.714–1.313	0.980
10:0	0.722	0.572–0.911	**0.047**	0.922	0.738–1.151	0.944
14:0	0.927	0.875–0.983	**0.047**	1.014	0.952–1.080	0.980
16:0	0.987	0.974–0.999	0.066	1.000	0.986–1.015	0.984
18:0	0.970	0.945–0.996	**0.047**	1.005	0.977–1.035	0.980
MUFA	0.995	0.989–1.001	0.140	0.996	0.989–1.003	0.664
16:1	0.956	0.844–1.083	0.482	1.149	0.995–1.327	0.347
18:1	0.995	0.988–1.001	0.140	0.996	0.989–1.003	0.664
n-6						
18:3	0.908	0.837–0.985	**0.047**	0.873	0.774–0.986	0.345
Model 3						
TSFA	0.991	0.985–0.998	**0.024**	0.998	0.990–1.006	0.919
8:0	0.665	0.455–0.972	**0.035**	1.036	0.752–1.426	0.919
10:0	0.755	0.594–0.959	**0.025**	0.988	0.787–1.241	0.919
14:0	0.931	0.877–0.987	**0.024**	1.017	0.955–1.084	0.919
18:0	0.965	0.941–0.991	**0.024**	0.996	0.966–1.027	0.919
n-6						
18:3	0.902	0.831–0.980	**0.024**	0.877	0.780–0.987	0.177

^a^Data are aORs, 95% CIs were calculated. Model 1: unadjusted model; Model 2: adjusted for age, sex; Model 3: adjusted for age, sex, race, BMI, education, ratio of family income to poverty, smoking, exercise and hypertension.

^b^*P* value corrected by FDR.

The association of dietary intakes of TFA, TSFA, MUFA, and PUFA quartile ranges with the risk of IGT is demonstrated in Table 4. NGT and quartile 1(Q1) were used as the reference group. The quartile range of the average dietary intake of the participants is depicted in supplementary table 1. In model 1, most of the dietary FAs in the range of Q3 and Q4 were negatively correlated with the risk of IGT. However, in model 2, SFAs including butanoic acid (4:0), hexanoic acid (6:0), octanoic acid (8:0), decanoic acid (10:0), and tetradecanoic acid (14:0) were negatively correlated with the risk of IGT. Further adjusted covariates in model 3, hexanoic acid (6:0) at 0.239–0.407 g/day was negatively correlated with the risk of IGT (OR = 0.747, 95% CI: 0.638–0.965, *P* = 0.024). Similarly, octanoic acid (8:0) at 0.3255–6.101 g/day, decanoic acid (10:0) at 0.610–4.611 g/day, and tetradecanoic acid (14:0) at 2.803–18.669 g/day were negatively associated with the risk of IGT.

**Table 4:** Association of different FAs ranges and risk of IGT in NHANES^a,b^.

FAs(g/d)	Q2^a^	*P*^b^	Q3^a^	*P*^b^	Q4^a^	*P*^b^
Model 1						
TFA	0.935 (0.747–1.169)	0.596	0.730 (0.592–0.901)	**0.014**	0.709 (0.575–0.874)	**0.007**
TSFA	0.902 (0.736–1.106)	0.386	0.770 (0.628–0.943)	**0.032**	0.634 (0.506–0.795)	**0.001**
4:0	0.882 (0.724–1.076)	0.304	0.712 (0.593–0.853)	**0.002**	0.668 (0.535–0.835)	**0.003**
6:0	0.875 (0.703–1.089)	0.314	0.697 (0.578–0.840)	**0.002**	0.714 (0.573–0.890)	**0.013**
8:0	0.902 (0.716–1.136)	0.443	0.758 (0.616–0.932)	**0.027**	0.634 (0.509–0.790)	**0.001**
10:0	0.923 (0.733–1.164)	0.555	0.796 (0.662–0.956)	**0.037**	0.619 (0.491–0.779)	**0.001**
12:0	0.818 (0.649–1.031)	0.135	0.702 (0.553–0.890)	**0.014**	0.685 (0.547–0.859)	**0.007**
14:0	0.970 (0.785–1.199)	0.804	0.743 (0.613–0.900)	**0.012**	0.598 (0.476–0.751)	**0.001**
16:0	0.889 (0.722–1.095)	0.352	0.842 (0.692–1.025)	0.135	0.658 (0.530–0.818)	**0.002**
18:0	0.892 (0.719–1.107)	0.370	0.890 (0.735–1.076)	0.314	0.650 (0.526–0.802)	**0.001**
MUFA	0.914 (0.720–1.161)	0.531	0.783 (0.632–0.969)	0.051	0.732 (0.587–0.913)	**0.020**
16:1	0.906 (0.744–1.103)	0.386	0.844 (0.687–1.036)	0.157	0.774 (0.626–0.955)	**0.039**
18:1	0.849 (0.667–1.080)	0.261	0.804 (0.648–0.996)	0.086	0.691 (0.556–0.858)	**0.006**
20:1	1.010 (0.797–1.279)	0.937	0.835 (0.689–1.011)	0.115	0.783 (0.627–0.977)	0.062
22:1	0.892 (0.724–1.098)	0.358	0.791 (0.653–0.957)	**0.037**	0.872 (0.715–1.062)	0.254
PUFA	0.799 (0.648–0.984)	0.066	0.791 (0.648–0.965)	**0.047**	0.781 (0.629–0.969)	0.051
n-6						
18:2	0.794 (0.642–0.981)	0.064	0.770 (0.631–0.939)	0.030	0.754 (0.603–0.944)	**0.036**
18:3	0.851 (0.711–1.019)	0.127	0.773 (0.629–0.951)	0.037	0.740 (0.596–0.917)	**0.021**
18:4	0.926 (0.752–1.141)	0.532	0.800 (0.636–1.006)	0.102	0.785 (0.652–0.946)	**0.031**
20:4	0.890 (0.720–1.102)	0.359	0.892 (0.728–1.093)	0.352	0.938 (0.762–1.155)	0.596
n-3						
20:5	0.828 (0.674–1.017)	0.123	0.736 (0.607–0.893)	**0.010**	0.822 (0.662–1.020)	0.127
22:5	0.839 (0.691–1.019)	0.127	0.762 (0.624–0.930)	**0.025**	0.711 (0.565–0.896)	**0.014**
22:6	0.979 (0.792–1.211)	0.858	1.033 (0.824–1.294)	0.804	1.053 (0.831–1.334)	0.713
Model 2						
TFA	0.954 (0.756–1.204)	0.742	0.795 (0.636–0.993)	0.138	0.802 (0.633–1.016)	0.174
TSFA	0.886 (0.713–1.102)	0.406	0.799 (0.642–0.994)	0.138	0.705 (0.545–0.910)	0.055
4:0	0.876 (0.715–1.074)	0.347	0.745 (0.611–0.908)	**0.040**	0.711 (0.564–0.897)	**0.040**
6:0	0.868 (0.686–1.098)	0.388	0.718 (0.587–0.879)	**0.020**	0.750 (0.593–0.948)	0.089
8:0	0.901 (0.713–1.139)	0.490	0.780 (0.621–0.979)	0.130	0.674 (0.537–0.846)	**0.020**
10:0	0.911 (0.713–1.165)	0.530	0.814 (0.672–0.985)	0.130	0.644 (0.506–0.821)	**0.020**
12:0	0.836 (0.655–1.066)	0.275	0.715 (0.554–0.924)	0.069	0.722 (0.572–0.912)	0.054
14:0	0.982 (0.788–1.225)	0.874	0.789 (0.64–0.967)	0.103	0.675 (0.530–0.858)	**0.020**
16:0	0.919 (0.735–1.149)	0.530	0.913 (0.740–1.128)	0.499	0.761 (0.597–0.971)	0.119
18:0	0.898 (0.718–1.124)	0.454	0.930 (0.757–1.142)	0.552	0.740 (0.582–0.940)	0.083
MUFA	0.931 (0.727–1.192)	0.628	0.851 (0.677–1.070)	0.296	0.8332 (0.649–1.069)	0.275
16:1	0.946 (0.779–1.149)	0.628	0.962 (0.781–1.185)	0.755	0.966 (0.760–1.227)	0.801
18:1	0.879 (0.687–1.125)	0.417	0.883 (0.700–1.114)	0.415	0.798 (0.625–1.020)	0.174
22:1	0.893 (0.716–1.113)	0.417	0.810 (0.664–0.989)	0.136	0.892 (0.727–1.094)	0.406
PUFA	0.818 (0.658–1.018)	0.174	0.857 (0.695–1.055)	0.275	0.862 (0.677–1.098)	0.382
n-6						
18:2	0.842 (0.670–1.058)	0.275	0.850 (0.687–1.050)	0.273	0.866 (0.674–1.113)	0.402
18:3	0.852 (0.713–1.019)	0.177	0.813 (0.653–1.011)	0.174	0.761 (0.603–0.960)	0.103
18:4	0.972 (0.788–1.200)	0.808	0.909 (0.714–1.159)	0.530	0.892 (0.731–1.089)	0.402
n-3						
20:5	0.886 (0.706–1.112)	0.415	0.820 (0.671–1.002)	0.157	0.915 (0.739–1.132)	0.504
22:5	0.842 (0.690–1.027)	0.193	0.826 (0.670–1.018)	0.174	0.805 (0.633–1.025)	0.177
Model 3						
4:0	0.895 (0.728–1.101)	0.367	0.784 (0.638–0.965)	0.064	0.755 (0.589–0.969)	0.069
6:0	0.862 (0.683–1.088)	0.289	0.747 (0.605–0.921)	**0.024**	0.762 (0.594–0.977)	0.069
8:0	0.927 (0.729–1.179)	0.577	0.791 (0.624–1.001)	0.088	0.701 (0.550–0.894)	**0.021**
10:0	0.911 (0.712–1.166)	0.531	0.840 (0.689–1.024)	0.127	0.662 (0.510–0.860)	**0.021**
14:0	0.997 (0.791–1.255)	0.976	0.810 (0.655–1.003)	0.088	0.688 (0.534–0.887)	**0.021**

^a^Data are adjusted OR (aOR), 95% CIs were calculated. Q quintile; Quintile 1 was the referent that was concealed. Model 1: unadjusted model; Model 2: adjusted for age, sex; Model 3: adjusted for age, sex, race, BMI, education, ratio of family income to poverty, smoking, exercise and hypertension.

^b^*P* value corrected by FDR.

The association between FA quartile ranges and the risk of T2DM is demonstrated in Table 5. Contrasting the IGT, in the three models, TFA was negatively correlated with the risk of T2DM. Nevertheless, the association between TSFA, MUFA, PUFA, and their subtypes and the risk of T2DM were not statistically significant in model 1, model 2, and model 3. Therefore, to determine the combined FAs that are associated with IGT and T2DM, the data were further analyzed by factor analysis. Analysis of the scree plot revealed that four factors had eigenvalues greater than the average eigenvalue Supplementary Fig. 1. The four factors described 78.010% of the total variability in the provided set of predictors. The rotated component matrix is indicated in Supplementary Table 2. Factor (F) 1 better represented the effect of SFAs whereas F4 represented octadecenoic acid (18:1), eicosenoic acid (20:1), octadecadienoic acid (18:2), and octadecatrienoic acid (18:3). The association between four factors and the risk of IGT and T2DM are presented in Table 6. Among the participants with IGT, F1 was negatively associated with the risk of IGT in model 1 and model 2 (OR = 0.872, 95% CI: 0.801–0.948, *P* = 0.005; OR = 0.889, 95% CI: 0.815–0.970, *P* = 0.031). No similar results were observed in Model 3. F4 was negatively associated with the risk of T2DM in the three models.

**Table 5:** Association of different FAs ranges and risk of T2DM in NHANES^a,b^.

FAs(g/d)	Q2^a^	*P*^b^	Q3^a^	*P*^b^	Q4^a^	*P*^b^
Model 1						
TFA	0.808 (0.616–1.061)	0.331	0.841 (0.654–1.083)	0.402	0.609 (0.468–0.793)	**0.016**
TSFA	0.937 (0.746–1.177)	0.824	0.954 (0.726–1.253)	0.857	0.722 (0.561–0.928)	0.108
4:0	0.920 (0.688–1.231)	0.824	0.968 (0.730–1.284)	0.908	0.875 (0.663–1.155)	0.613
6:0	0.982 (0.752–1.281)	0.931	1.046 (0.803–1.362)	0.857	0.944 (0.710–1.256)	0.855
8:0	0.981 (0.766–1.257)	0.931	1.063 (0.821–1.375)	0.826	0.897 (0.705–1.142)	0.653
10:0	1.069 (0.834–1.371)	0.824	1.045 (0.803–1.358)	0.857	0.860 (0.665–1.114)	0.473
12:0	1.084 (0.834–1.410)	0.824	1.053 (0.815–1.361)	0.855	0.847 (0.659–1.090)	0.416
14:0	1.076 (0.866–1.338)	0.824	1.002 (0.781–1.285)	0.987	0.861 (0.669–1.110)	0.473
16:0	0.987 (0.757–1.285)	0.948	0.866 (0.686–1.093)	0.459	0.739 (0.579–0.945)	0.108
18:0	0.955 (0.746–1.223)	0.857	0.924 (0.725–1.178)	0.824	0.794 (0.619–1.017)	0.222
MUFA	0.936 (0.722–1.214)	0.826	0.770 (0.592–1.002)	0.197	0.722 (0.557–0.935)	0.108
16:1	0.972 (0.748–1.263)	0.908	0.813 (0.613–1.078)	0.372	0.939 (0.728–1.211)	0.826
18:1	0.994 (0.744–1.328)	0.980	0.839 (0.656–1.072)	0.382	0.711 (0.557–0.909)	0.099
20:1	0.882 (0.656–1.184)	0.676	0.815 (0.625–1.062)	0.333	0.702 (0.521–0.947)	0.108
22:1	0.965 (0.729–1.276)	0.905	0.842 (0.634–1.118)	0.462	1.070 (0.801–1.429)	0.826
PUFA	0.808 (0.627–1.040)	0.271	0.704 (0.514–0.964)	0.140	0.635 (0.466–0.865)	0.092
n-6						
18:2	0.787 (0.611–1.014)	0.222	0.691 (0.514–0.929)	0.108	0.640 (0.462–0.886)	0.099
18:3	0.817 (0.600–1.112)	0.416	0.772 (0.570–1.045)	0.271	0.642 (0.448–0.921)	0.108
18:4	0.733 (0.540–0.996)	0.192	0.705 (0.525–0.945)	0.108	0.735 (0.556–0.971)	0.140
20:4	1.066 (0.845–1.345)	0.824	0.817 (0.609–1.097)	0.402	1.081 (0.820–1.425)	0.824
n-3						
20:5	0.753(0.594–0.953)	0.108	0.646 (0.489–0.854)	0.074	0.783 (0.590–1.040)	0.271
22:5	0.976 (0.758–1.257)	0.918	0.778 (0.596–1.016)	0.222	0.748 (0.563–0.994)	0.192
22:6	1.079 (0.842–1.382)	0.824	0.889 (0.696–1.136)	0.613	1.266 (0.980–1.635)	0.222
Model 2						
TFA	0.826 (0.619–1.102)	0.709	0.929 (0.711–1.215)	0.933	0.703 (0.534–0.926)	**0.012**
Model 3						
TFA	0.902 (0.671–1.214)	0.497	0.959 (0.720–1.277)	0.776	0.714 (0.532–0.959)	**0.025**

^a^Data are aOR, 95% CIs were calculated. Q, quintile; Quintile 1 was the referent that was concealed. Model 1: unadjusted model; Model 2: adjusted for age, sex; Model 3: adjusted for age, sex, race, BMI, education, ratio of family income to poverty, smoking, exercise and hypertension.

^b^*P* value corrected by FDR.

**Table 6:** Prediction of IGT and T2DM events estimated for individual factors^a,b^.

	IGT		T2DM	
	OR (95%CI)^a^	*P*^b^	OR (95%CI)^a^	*P*^b^
Model 1				
F1	0.872 (0.801–0.948)	**0.005**	0.965 (0.888–1.049)	0.674
F2	0.976 (0.894–1.066)	0.595	1.004 (0.891–1.131)	0.949
F3	0.958 (0.893–1.026)	0.294	1.037 (0.931–1.155)	0.674
F4	0.927 (0.857–1.002)	0.113	0.823 (0.717–0.944)	**0.021**
Model 2				
F1	0.889 (0.815–0.970)	**0.031**	0.990 (0.911–1.076)	0.823
F2	0.984 (0.896–1.081)	0.741	1.014 (0.899–1.144)	0.823
F3	1.042 (0.959–1.133)	0.438	1.169 (1.029–1.328)	**0.033**
F4	0.929 (0.854–1.011)	0.173	0.826 (0.718–0.951)	**0.030**
Model 3				
F1	0.907 (0.829–0.993)	0.100	1.016 (0.934–1.105)	0.712
F2	1.008 (0.918–1.107)	0.869	1.049 (0.936–1.176)	0.550
F3	1.014 (0.934–1.101)	0.869	1.114 (0.974–1.273)	0.229
F4	0.920 (0.847–1.000)	0.100	0.824 (0.715–0.949)	**0.029**

^a^Data are aORs, 95% CIs were calculated. Model 1: unadjusted model; Model 2: adjusted for age, sex; Model 3: adjusted for age, sex, race, BMI, education, ratio of family income to poverty, smoking, exercise and hypertension.

^b^*P* value corrected by FDR.

## Discussion

Our research yields the following findings: a) In the American population, TFA was generally associated with a higher risk of T2DM, b) the correlations between the risk of IGT and T2DM for various FAs and dose ranges of the same FA vary, c) SFA plays a distinct role in both T2DM and IGT, SFA subtypes were negatively correlated to IGT but not to T2DM, d) the protective associations between F4 and the risk of T2DM were driven by octadecenoic acid (18:1), eicosenoic acid (20:1), octadecadienoic acid (18:2), and octadecatrienoic acid (18:3).

In our study, the negative relationship between TSFAs and the risk of IGT should not be overlooked. However, TSFA was not statistically associated with T2DM, and the result is consistent with three prospective studies [13–15]. Consistent with the results of the latest systematic review of 23 prospective cohort studies, our study observed a weak linear association between dietary TFA and the risk of T2DM [16]. Another 14-year cohort study of nurses revealed that TSFA intake was not correlated with the risk of T2DM among females [13]. Two prospective studies also observed no significant correlation between TFA or specific types of FA intakes and the risk of T2DM. These findings were limited by the confusion of non-dietary factors, dietary assessment bias, and the number of focusses [14, 15, 17]. A prospective epidemiological study demonstrated no correlation between total SFA and T2DM, which was consistent with our findings [18].

In several prospective studies, the total MUFA and PUFA intakes were inversely associated with the odds of T2DM [19–21]. The sheltering effect of FAs on metabolic diseases is partly owing to the regulation of microRNAs by modulating gene expression [22]. In our study, the association between individual unsaturated FAs and T2DM was not significant. Factor analysis revealed that four combined unsaturated FAs were negatively correlated with T2DM. Dietary oil contains a large number of unsaturated FAs, of which octadecenoic acid (18:1), octadecadienoic acid (18:2), and octadecatrienoic acid (18:3) are the most abundant. In contrast, eicosenoic acid (20:1) has achieved less focus. These results suggest that we should focus on these specific FAs to improve the risk of IGT and T2DM rather than concentrating on general MUFA or PUFA. This could also be the cause of the contradictory results of many studies on the impact of MUFA and PUFA on the risk of T2DM.

Our study has a few notable advantages. First, we excluded individuals who were previously diagnosed with T2DM to remove the Neyman bias. Since the NHANES data can identify patients with undiagnosed T2DM, and more than a third of the T2DM groups were overlooked, we only included newly diagnosed participants who did not change their dietary patterns. Second, our study fixed the flaw that dietary FAs cannot be quantified in previous studies. Along with the precise classification of FAs, we also evaluated how different dose ranges of FA subtypes were related to IGT and T2DM. Our study partially corrects the fact that there is a gap between blood and dietary FAs, which makes it difficult to recommend adequate amounts of FAs in the diet. This study also has some limitations. Since it was a cross-sectional study, no definitive causal relationship was established, and prospective and experimental studies are required to verify these associations. The 24-hour dietary recall interview method only reflected the short-term dietary FA exposure level. Since the food frequency questionnaires could not be obtained to reflect the long-term dietary FAs intake level, there may be a certain degree of bias in the estimation of dietary FAs. Additionally, although the overall sample size was optimal, the sample size of IGT and T2DM was relatively small.

## Conclusions

Dietary TFA was negatively associated with T2DM in American adult populations. Unsaturated FAs with high proportions in natural oils may reduce the risk of T2DM. More research is required to determine the role of FAs in the development of diabetes.

## Supplementary information

Legend for Figure 1 supplementary: Scree plot representing the eigenvalues versus the factor numbers.

Figure 1 supplementary: Scree plot representing the eigenvalues versus the factor numbers.

Table1 supplementary. The quartiles range of average dietary intake of the participants

Table 2 supplementary. Standardized loadings (pattern matrix) based upon correlation matrix

## Data Availability

All data used in the present study were provided by the National Health and Nutrition Examination Survey (NHANES) and could be downloaded at http://www.cdc.gov/nchs/nhances/.
