# Supplementary material for: Association between fatty acids and the risk of impaired glucose tolerance and type 2 diabetes mellitus in American adults: NHANES 2005−2016
Source: Nutr Diabetes. 2023 May 1;13:8. doi: 10.1038/s41387-023-00236-4 (PMC10151340; doi:10.1038/s41387-023-00236-4)
Supplement: Supplementary file 1 — Legend for Figure 1 supplementary: Scree plot representing the eigenvalues versus the factor numbers. [file 41387_2023_236_MOESM1_ESM.docx]

**Figure 1** **supplementary:** Scree plot representing the eigenvalues versus the factor numbers.
