# Supplementary figures and images for: Association between fatty acids and the risk of impaired glucose tolerance and type 2 diabetes mellitus in American adults: NHANES 2005−2016
Source: Nutr Diabetes. 2023 May 1;13:8. doi: 10.1038/s41387-023-00236-4 (PMC10151340; doi:10.1038/s41387-023-00236-4)

**Scree plot**

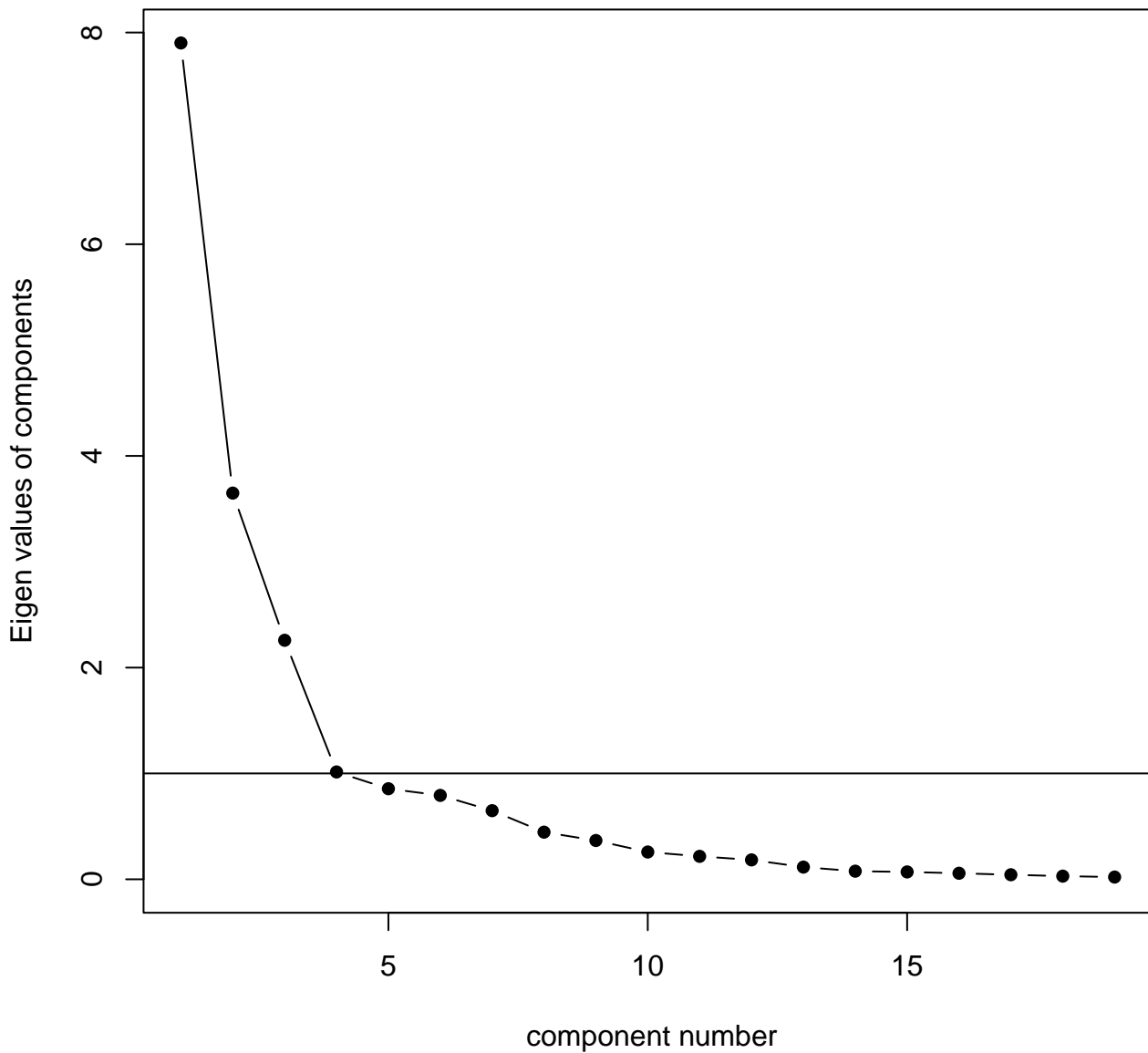

Supplement: Supplementary file 2 — Figure 1 supplementary: Scree plot representing the eigenvalues versus the factor numbers. [file 41387_2023_236_MOESM2_ESM.pdf]
