# Supplementary material for: Association between fatty acids and the risk of impaired glucose tolerance and type 2 diabetes mellitus in American adults: NHANES 2005−2016
Source: Nutr Diabetes. 2023 May 1;13:8. doi: 10.1038/s41387-023-00236-4 (PMC10151340; doi:10.1038/s41387-023-00236-4)
Supplement: Supplementary file 3 — Table1 supplementary. The quartiles range of average dietary intake of the participants [file 41387_2023_236_MOESM3_ESM.docx]

**Table1 supplementary.** The quartiles range of average dietary intake of the participants

| **Variables** | **Q1** | **Q2** | **Q3** | **Q4** |
| --- | --- | --- | --- | --- |
| TFA | 0.630~50.328 | 50.328~70.980 | 70.980~96.663 | 96.663~426.925 |
| TSFA | 0.010~15.589 | 15.589~22.757 | 22.757~32.031 | 32.031~137.958 |
| 4:0 | 0.000~0.203 | 0.203~0.407 | 0.407~0.702 | 0.702~4.544 |
| 6:0 | 0.000~0.121 | 0.121~0.239 | 0.239~0.407 | 0.40725~3.014 |
| 8:0 | 0.000~0.100 | 0.100~0.190 | 0.190~0.326 | 0.3255~6.101 |
| 10:0 | 0.000~0.196 | 0.196~0.369 | 0.369~0.610 | 0.610~4.611 |
| 12:0 | 0.000~0.255 | 0.255~0.498 | 0.498~0.904 | 0.904~33.674 |
| 14:0 | 0.000~1.027 | 1.027~1.769 | 1.769~2.803 | 2.803~18.669 |
| 16:0 | 0.000~8.771 | 8.771~12.477 | 12.477~17.410 | 17.410~120.117 |
| 18:0 | 0.000~3.889 | 3.889~5.725 | 5.725~8.081 | 8.081~61.594 |
| MUFA | 0.072~17.798 | 17.798~25.374 | 25.374~34.860 | 34.860~174.477 |
| 16:1 | 0.000~0.640 | 0.640~0.999 | 0.999~1.484 | 1.484~10.161 |
| 18:1 | 0.000~16.517 | 16.517~23.667 | 23.6665~32.8 | 32.800~211.699 |
| 20:1 | 0.000~0.140 | 0.140~0.222 | 0.222~0.345 | 0.345~3.872 |
| 22:1 | 0.000~0.003 | 0.003~0.011 | 0.011~0.034 | 0.034~7.030 |
| PUFA | 0.005~10.606 | 10.606~15.555 | 15.555~22.114 | 22.114~120.595 |
| n-6 |  |  |  |  |
| 18:2 | 0.000~9.292 | 9.292~13.661 | 13.661~19.686 | 19.686~112.755 |
| 18:3 | 0.000~0.923 | 0.923~1.367 | 1.367~1.990 | 1.990~16.509 |
| 18:4 | 0.000~0.000 | 0.000~0.002 | 0.002~0.010 | 0.010~0.62 |
| 20:4 | 0.000~0.072 | 0.072~0.124 | 0.124~0.198 | 0.198~1.148 |
| n-3 |  |  |  |  |
| 20:5 | 0.000~0.004 | 0.004~0.009 | 0.009~0.022 | 0.022~2.687 |
| 22:5 | 0.000~0.008 | 0.008~0.016 | 0.016~0.029 | 0.029~0.919 |
| 22:6 | 0.000~0.008 | 0.008~0.029 | 0.029~0.074 | 0.074~4.078 |
