# Supplementary material for: Association between fatty acids and the risk of impaired glucose tolerance and type 2 diabetes mellitus in American adults: NHANES 2005−2016
Source: Nutr Diabetes. 2023 May 1;13:8. doi: 10.1038/s41387-023-00236-4 (PMC10151340; doi:10.1038/s41387-023-00236-4)
Supplement: Supplementary file 4 — Table 2 supplementary. Standardized loadings (pattern matrix) based upon correlation matrix [file 41387_2023_236_MOESM4_ESM.docx]

| **FAs(g/d)** | **F1** | **F2** | **F3** | **F4** |
| --- | --- | --- | --- | --- |
| 4:0 | **0.85** | -0.01 | 0.37 | 0.00 |
| 6:0 | **0.87** | -0.01 | 0.31 | 0.02 |
| 8:0 | **0.90** | 0.03 | -0.04 | 0.16 |
| 10:0 | **0.95** | 0.01 | 0.19 | 0.11 |
| 12:0 | **0.73** | 0.04 | -0.23 | 0.26 |
| 14:0 | **0.89** | 0.02 | 0.39 | 0.13 |
| 16:0 | **0.54** | 0.01 | **0.68** | 0.45 |
| 18:0 | **0.53** | -0.01 | **0.68** | 0.41 |
| 16:1 | 0.29 | 0.09 | **0.81** | 0.26 |
| 18:1 | 0.29 | 0.03 | **0.61** | **0.66** |
| 20:1 | 0.06 | 0.44 | 0.32 | **0.60** |
| 22:1 | 0.01 | **0.56** | -0.02 | 0.09 |
| 18:2 | 0.12 | 0.04 | 0.30 | **0.89** |
| 18:3 | 0.17 | 0.06 | 0.16 | **0.82** |
| 18:4 | 0.08 | **0.69** | 0.10 | 0.03 |
| 20:4 | -0.01 | 0.24 | **0.67** | 0.23 |
| 20:5 | -0.02 | **0.92** | -0.02 | 0.02 |
| 22:5 | -0.02 | **0.82** | 0.21 | 0.04 |
| 22:6 | -0.04 | **0.94** | 0.02 | 0.04 |

**Table 2 supplementary.** Standardized loadings (pattern matrix) based upon correlation matrix

rotated method: maximum variance method
